# Effects of tailored interventions for anxiety management in choking-susceptible performing artists: a mixed-methods collective case study

**DOI:** 10.3389/fpsyg.2023.1164273

**Published:** 2023-05-18

**Authors:** Veronika J. Lubert, Sanna M. Nordin-Bates, Peter Gröpel

**Affiliations:** ^1^Department of Occupational, Economic, and Social Psychology, Faculty of Psychology, University of Vienna, Vienna, Austria; ^2^Department of Physical Activity and Health, Swedish School of Sport and Health Sciences, Stockholm, Sweden; ^3^Department of Sport Science, Center for Sport Science and University Sports, University of Vienna, Vienna, Austria

**Keywords:** performance anxiety, performing arts, performance under pressure, psychological interventions, tailored interventions, choking, mixed-methods

## Abstract

**Introduction:**

Not being able to manage performance anxiety and subsequently experiencing a decline in performance have been called “choking under pressure”. High trait anxiety and fear of negative evaluation, as well as low self-efficacy or self-confidence, can put performers especially at risk of experiencing choking. This study, therefore, examined the effects of psychological choking interventions tailored to “choking-susceptible” performing artists individually in a coaching setting.

**Methods:**

We conducted a mixed-methods (QUANT + QUAL) collective case study with nine performing artists, who each received five individual coaching sessions. The tailored choking interventions comprised acclimatization training, goal setting, and pre-performance routines, including elements such as imagery, self-talk, and relaxation techniques. Before and after the 10-week intervention phase, they filled in questionnaires on trait performance anxiety, fear of negative evaluation, and self-efficacy, performed in front of a jury, and were interviewed about their experiences. Transcripts of interviews and coaching sessions were analyzed using thematic analysis. Heart rate measurements, weekly performance videos, and expert evaluations were also part of our comprehensive data.

**Results:**

Quantitative data showed reductions in performance anxiety and fear of negative evaluation, and increases in self-efficacy and performance quality, from before to after the intervention phase. Most participants also had a lower heart rate when performing for the jury. Themes from qualitative analysis comprised managing nervousness and feeling more relaxed, becoming more self-confident, satisfaction with artistic and mental performance, feeling good and enjoying performing, and general positive effects.

**Conclusion:**

Tailoring psychological interventions may provide several benefits for choking-susceptible performing artists.

## 1. Introduction

Performing artists often have to perform in front of an audience, which may lead them to experience high pressure and anxiety, eventually harming their performance. Psychologists have called this “choking under pressure,” a phenomenon that refers to performing worse than expected despite high skills and motivation to perform well (Baumeister, 1984). Choking has been mostly studied in sports (Mesagno and Beckmann, 2017; Gröpel and Mesagno, 2019), yet the drop in performance under pressure is also relevant in the performing arts (Hays, 2017). Consequently, and due to the similarities shared with sports (Mesagno et al., 2016), researchers have begun transferring sport psychological interventions into the field of performing arts and specifically testing them for performance under pressure (Tief and Gröpel, 2021; Lubert and Gröpel, 2022). However, compared to interventions designed to prevent choking in athletes (Gröpel and Mesagno, 2019), the observed benefits were fewer than those found in sports, as there was no effect on expert-rated performance quality. We thus need a deeper understanding of how sport psychological interventions may be specifically tailored to the performing arts in the pursuit of more pronounced performance-related benefits.

One issue when designing choking intervention studies is the presence of pressure. Participants must feel, and perform under, pressure to validate an intervention, which has been typically induced by setting up a competition, providing rewards, or performing in front of an audience (Gröpel and Mesagno, 2019). Alternatively, or additionally, researchers have studied “choking-susceptible” persons (i.e., individuals likely to experience choking), as they are expected to benefit most from choking interventions (Mesagno et al., 2008, 2009). Such persons are typically characterized by high trait anxiety, fear of negative evaluation (FNE), self-consciousness, or low self-efficacy (Wang et al., 2004; Mesagno et al., 2021).

Performance anxiety as a specific kind of trait anxiety can be defined as persisting worries about and heightened physiological arousal in relation to public performance, which may lead to a decline of performance skills in the presence of an audience (Salmon, 1990; Kenny, 2011). Studies with musicians and dancers highlight that the cognitive dimension of anxiety (e.g., worry) is often perceived as more debilitative to performance than the somatic dimension (e.g., “butterflies” in the stomach; Miller and Chesky, 2004; Walker and Nordin-Bates, 2010). Compared to individuals low in trait anxiety, those with high trait anxiety may be more prone to perceive evaluative situations as threatening (Byrne and Eysenck, 1995). Consequently, individuals high in trait anxiety experience higher levels of stress and state anxiety and also perform worse under pressure, than individuals low in trait anxiety (Kubzansky and Stewart, 1999; Schlotz et al., 2006). High trait anxiety is, therefore, considered to be one of the best predictors of choking (Wang et al., 2004).

It has been suggested that performance anxiety increases due to concerns about self-presentation (Mesagno et al., 2012). That is, performers strive to create positive images of themselves in the presence of an audience, but if they doubt their competence, they become anxious about their public image. A link between choking and concerns about self-presentation can be the performers' tendency to focus on the possibility that the audience will evaluate them as a social object, which elevates their FNE (Mesagno et al., 2012). Indeed, it has been demonstrated that high FNE leads to choking, whereas low FNE does not (Mesagno et al., 2012).

Finally, a performer's confidence in their ability to self-regulate themselves and their environment has been identified as a protective characteristic against the debilitative effects of anxiety. With strong self-confidence, anxiety can indeed be perceived as facilitative for performance (Hanton et al., 2004). This has also been shown with professional dancers: self-confidence may protect against debilitative performance anxiety, as it helps to feel in control and reinterpret anxiety symptoms (Walker and Nordin-Bates, 2010). Whereas self-confidence is a generic term that indicates “strength of belief but does not necessarily specify what the certainty is about”, self-efficacy means the “belief in one's agentive capabilities, that one can produce given levels of attainment” regarding a specific skill or task (Bandura, 1997, p. 382). Self-confidence is a more commonly used term in everyday language, yet may often mean self-efficacy in the sense of one's confidence in a particular ability. Throughout the article, we apply the respective terms as used in the questionnaires or in participants' quotes. In music, self-efficacy for performing was shown to be a strong predictor of performance quality in both self-evaluations and jury evaluations (Ritchie and Williamon, 2012).

In sum, individuals high in trait performance anxiety and FNE and low in self-efficacy may be especially susceptible to choking. Indeed, musicians high in trait performance anxiety benefited from choking interventions, whereas those low in trait anxiety did not, presumably because the experience of pressure was only at a medium level (Lubert and Gröpel, 2022). Consequently, to shed more light on the effectiveness of choking interventions in the performing arts, we need to sample choking-susceptible artists and let them perform under pressurized conditions.

Another issue in intervention research is “person-treatment matching,” which refers to matching intervention strategies with the characteristics and needs of an individual. Several researchers have emphasized the importance of tailoring interventions to performers' specific needs (Lidor and Mayan, 2005; Cotterill et al., 2010), as tailored interventions are likely to induce more visible benefits than generic “one-size-fits-all” interventions and might also increase the individuals' willingness to apply the intervention (Mesagno et al., 2021). This could be especially relevant with performing artists: some dancers who felt unable to manage their anxiety believed that psychological interventions would not help them, as everybody has different needs and coping styles (Walker and Nordin-Bates, 2010). The implementation of interventions inspired by sport psychology may therefore be challenging for artists and requires a domain-sensitive, holistic approach (Pecen et al., 2016). Pioneering work has been done in two case studies with musicians who received tailored psychological skills training, which showed facilitative effects on different psychological and performance aspects (Hatfield, 2016; Hatfield and Lemyre, 2016). Hence, a tailored approach could provide valuable insights into informed research and practical applications in music, dance, and acting.

Choking theories imply that performers fail under pressure because of increased anxiety and subsequent maladaptive attention (Mesagno and Beckmann, 2017). Psychologists have, therefore, developed interventions aimed to adapt performers to pressure and improve their concentration (Gröpel and Mesagno, 2019). These interventions comprise, but are not limited to, acclimatization training, pre-performance routines, and goal setting. Acclimatization training refers to practicing under mild anxiety conditions, which can either be a kind of behavioral exposure (e.g., the presence of an audience) or a simulation of expected demands and consequences of an individual's performance (e.g., rewards, punishments, and perceived evaluation by coaches). Evidence shows moderate-to-large effects of acclimatization training on posttest performance under pressure, indicating its effectiveness in familiarizing performers with pressure (Low et al., 2021). A pre-performance routine (PPR) is a set of cognitive and behavioral elements a performer systematically engages in prior to performance execution (Moran, 1996). The main function of a PPR is to enhance concentration by directing attention to task-specific cues and minimizing internal or external distractions (Cotterill, 2010). It typically comprises task-specific motor actions, such as when a dancer marks parts of the choreography with their hands. Notably, such behavioral elements are often combined with one or more mental strategies, e.g., with imagery (e.g., visualization of successful performance), self-talk (e.g., positive self-instructions), or relaxation elements (e.g., a couple of deep breaths before performing). Meta-analyses show moderate-to-large effects of PPRs in sports and support the benefits of both behavioral and mental elements for performance under pressure (Rupprecht et al., 2021). Finally, setting process goals has often been used in sports to facilitate task-relevant attention when performing a specific skill (Weinberg and Butt, 2014). A process goal is focused on the key steps underpinning the performance (e.g., a cellist maintaining a smooth bow stroke) and is fully controllable by the individual, as opposed to an outcome goal which is focused on the desired end result (e.g., winning a role or a prize) and thus often not within the performer's full control. Researchers have documented significant benefits of goal setting for sports, with process goals having the largest effects on performance and self-efficacy (Williamson et al., 2022). These interventions are not limited to sports, but have the potential to help performing artists as well.

Previous intervention research in the performing arts has had a stronger focus on reducing anxiety than on improving performance, and is unevenly distributed between domains: while there are numerous studies with musicians, there are very few with dancers and actors. In the past decade, several researchers in music performance have investigated some of the interventions described above within extensive psychological skills training (PST) similar to or based on PST interventions in sports psychology. These training programs consisted of different combinations of cognitive restructuring, behavioral exposure to performances, identification of strengths, goal-setting, imagery, practice strategies, arousal regulation, and relaxation, but were not specifically focused on high-pressure performance settings. Studies showed that PST interventions had a positive effect on music performance anxiety and performance quality in young musicians (Braden et al., 2015), aspiring professionals (Spahn et al., 2016), and musicians of all levels (Hoffman and Hanrahan, 2012). PST interventions for musicians that also included PPRs were associated with higher self-efficacy and more control over or reduction of anxiety after the training, but measures of performance quality were often either lacking (Osborne et al., 2014; Hatfield, 2016; Kinne, 2016) or provided inconclusive results (Kageyama, 2007; Clark and Williamon, 2011). In contrast, a recent study showed improvements in both performance quality and anxiety after a PST intervention including PPRs, goal-setting, positive self-talk, imagery, memorization, arousal regulation, and relaxation (Cohen and Bodner, 2019). In dance, PST was explored in a pilot study aiming at injury prevention, rather than anxiety management for performance under pressure (Skvarla and Clement, 2019).

Remarkably, none of the previous experimental studies on PST were focused on individuals with high trait performance anxiety. Instead, exploratory studies targeted at highly anxious performing artists have so far only investigated acceptance and commitment therapy or coaching (ACT or ACC). In music, a case study was undertaken with a highly anxious violinist, who was guided toward mindfulness and acceptance of her unwanted anxiety symptoms through ACT (Juncos and Markman, 2016). Her performance quality improved after the intervention, and even though symptom reduction had not been the study aim, performance anxiety was reduced as well. However, whether the pre- and post-intervention performances “to a small audience” (Juncos and Markman, 2016, p. 8) were truly perceived as pressure-inducing remains unclear. A study with six highly anxious performing arts students receiving group ACC indicated reduced performance anxiety after the intervention, but there was no effect on performance, and the performance setting was again likely no high-pressure situation (Mahony et al., 2022).

Taken together, evidence for the effectiveness of interventions similar to those tested to prevent choking in sports is generally promising. However, studies have not yet been focused on improving performance quality in high-pressure performance settings or on performing artists with high performance anxiety. Exploratory interventions targeted at highly anxious individuals show mixed evidence and were likely not conducted in high-pressure situations. With this study, we, therefore, intend to advance the knowledge transfer between sports psychology and the performing arts of music, dance, and acting by sampling choking-susceptible performing artists and tailoring acclimatization training, goal-setting, and/or PPRs, including elements such as imagery, self-talk, or relaxation techniques, to their needs in relation to performance under pressure. Our aim is to investigate the effects of these interventions on participants' performance quality and the key personal characteristics that make them susceptible to choking (anxiety, FNE, and low self-efficacy or self-confidence), and to examine their experiences with the interventions using a collective, mixed-methods case study design.

## 2. Materials and methods

### 2.1. Design and approach

This collective case study is embedded in a theoretical approach of critical realism: by believing in a reality independent of our construal of it, and in the notion that all our construed knowledge about this reality is incomplete and interpretative, we adopt a combination of ontological realism with epistemological relativism (Easton, 2010; Braun and Clarke, 2021). A collective case study extends an instrumental case study to several cases, which is justified by an interest in a phenomenon rather than the intrinsic interest in just one particular case (Stake, 1998). In choosing a collective case study, we aimed for an in-depth, multi-perspectival investigation of implementing and evaluating tailored interventions with a small number of highly anxious performing artists from different genres to foster a better understanding, or potentially better theorizing, for an even larger case collection in future research (Stake, 1998; Simons, 2009; Hodge and Sharp, 2019). Inspired by a similar approach with choking-susceptible athletes (Mesagno et al., 2008), we conceptualized this pre–post evaluation of intervention effects as a parallel mixed-methods design with simultaneous collections of qualitative and quantitative data (Kuckartz, 2014). We consider both qualitative and quantitative approaches as equally important to address our research questions (QUANT + QUAL) and thus interleaved them dynamically and interactively in every phase of the study (Kuckartz, 2014). Consequently, it appears appropriate to apply quality judgment criteria for the quantitative and qualitative aspects of the study separately, while also seeing them as discrete and bounded (Sparkes, 2015).

### 2.2. Participants

Participants were recruited from a renowned performing arts university in Austria. We selected them based on both qualitative and quantitative considerations. Musicians, dancers, and actors were invited via email to sign up via a link to an online questionnaire if they felt they were generally strongly affected by performance anxiety. Twenty-one performing arts students considered this to apply to them by accepting the invitation online. They gave informed consent according to the Declaration of Helsinki and filled in demographic as well as three psychological questionnaires for the subsequent quantitative identification of choking-susceptible participants. Ten participants were then purposively selected (see below). Due to COVID-19 and uncertainties about performance schedules at the time of selection, some of those originally chosen withdrew their participation. One musician did not wish to apply psychological interventions and thus received a different kind of coaching. Consequently, she was excluded from the analyses in this article. The final sample (eight females and one male) included six musicians, two dancers, and one actress. They were 20–26 years old (*M* = 23.3, *SD* = 2.2), had on average 15.2 years (*SD* = 2.9) of experience in their respective performance domain, and practiced or trained their skills for an average of 26.1 h (*SD* = 13.8) per week. Table 1 shows their demographic data. All names are pseudonyms. The study was approved by the Institutional Review Board of the first author's institution (#2021/S/004) and ran from April to July 2021. Participants received 100 EUR for their participation.

**Table 1:** Demographic data of each participant.

**Pseudonym**	**Performance major**	**Genre**	**Age**	**Gender**	**Nationality**	**Years in the domain**	**Hours/week**	**Study level and semester**
Anne	Dance	Contemporary	24	Female	Austrian	18	38	B7
Bianca	Trumpet	Classical	20	Female	Austrian	10	14	B1
Coco	Dance	Contemporary	22	Female	Canadian	18	24	B6
Julia	Accordion	Classical	20	Female	German	12	28	B4
Lucy	Acting	Stage acting/ musical	26	Female	German	15	56	B8
Mia	Violin	Baroque	23	Female	Hungarian	19	15	B7
Tom	Trumpet	Jazz	25	Male	German	15	28	B4
Vivi	Clarinet	Classical	25	Female	Austrian	15	17	M2
Zoe	Voice	Jazz	25	Female	Turkish	15	15	B2

B, bachelor's program; M, master's program.

### 2.3. Materials and procedure

**Figure 1:**
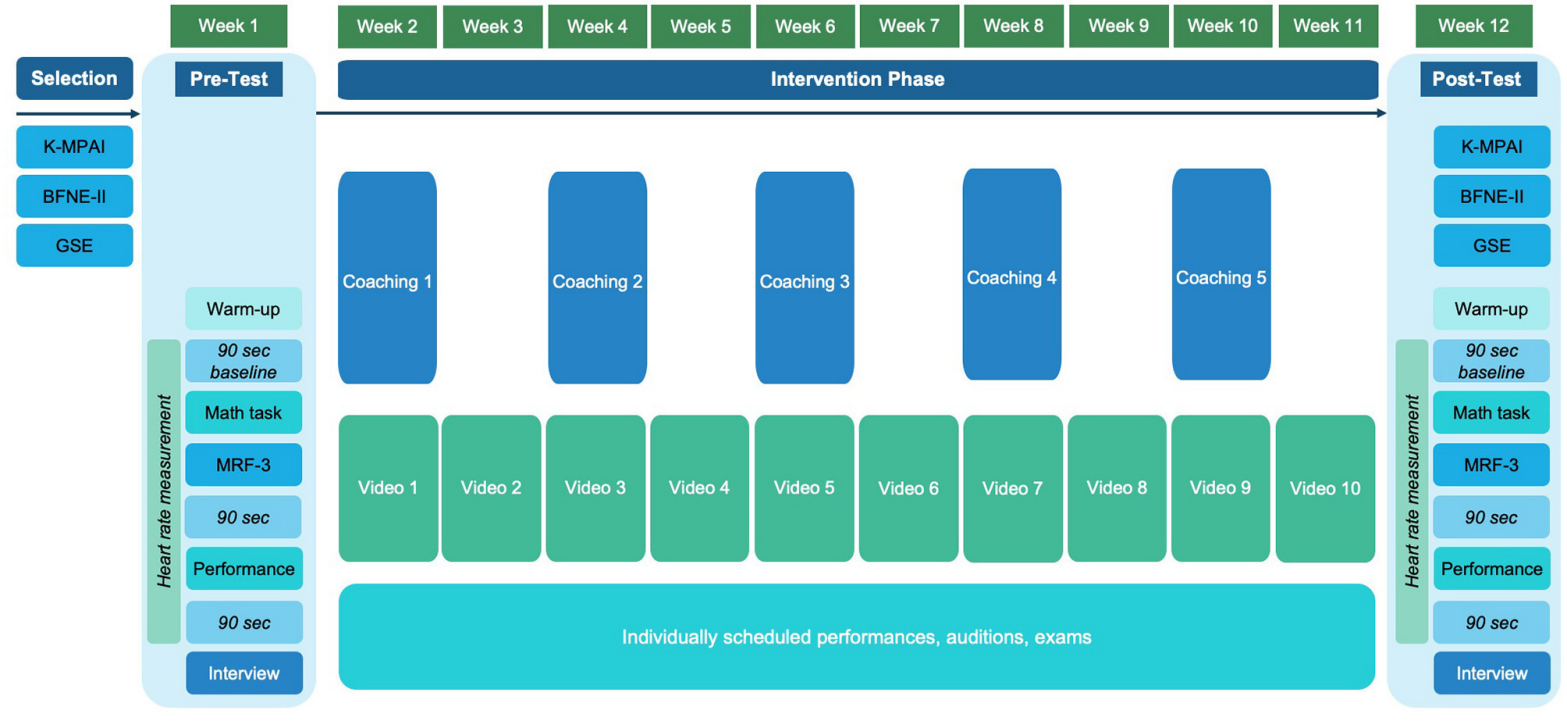
Design and procedure. K-MPAI, Kenny Music Performance Anxiety Inventory; BFNE-II, Brief Fear of Negative Evaluation Scale-Revised; GSE, General Self-Efficacy Scale; MRF-3, Mental Readiness Form.

The study consisted of four distinct phases: (1) selection, (2) pretest, (3) intervention, and (4) posttest (Figure 1). Materials, interviews, and coaching sessions were in German and English, depending on the participants' language preferences.

During *selection*, all invited artists completed questionnaires on trait anxiety, FNE, and self-efficacy. Trait performance anxiety was measured with a short version of the Kenny Music Performance Anxiety Inventory (K-MPAI; Kenny, 2011). We included 19 items from three dimensions: proximal somatic anxiety, worry/dread, and focus on self/other scrutiny. An example item is: “Prior to, or during a performance, I get feelings akin to panic”. A 7-point scale ranging from 0 (*strongly disagree*) to 6 (*strongly agree*) was employed. The K-MPAI is widely used, and its reliability and validity have been supported by evidence from numerous studies (e.g., Chang-Arana et al., 2018). Fear of negative evaluation was assessed with the Brief Fear of Negative Evaluation Scale-Revised (Carleton et al., 2006) and its German translation (Reichenberger et al., 2016). The scale consists of 12 items, such as “I am afraid that others will not approve of me”, and items were answered on a 5-point scale from 1 (*not at all characteristic of me*) to 5 (*extremely characteristic of me*). Psychometric testing of the BFNE-II has shown acceptable psychometric properties (Carleton et al., 2006; Reichenberger et al., 2016). Finally, self-efficacy was measured using the English and German versions of the General Self-Efficacy Scale (GSE; Schwarzer and Jerusalem, 1995, 2003). Their 10 items have a 4-point scale ranging from 1 (*not at all true*) to 4 (*exactly true*), an example being “I can usually handle whatever comes my way”. Reliability and validity for this scale have been extensively established (Schwarzer and Jerusalem, 1995, 2003). Artists with the highest percentile rank above the norm in trait performance anxiety and FNE, and the lowest percentile ranks below the norm in self-efficacy, were selected to participate.

In the *pretest*, participants were exposed to a psychosocial stressor using a procedure similar to the Trier Social Stress Test^1^ (TSST; Kirschbaum et al., 1993): participants were asked to perform a mental arithmetic task in front of a jury, followed by performing audition excerpts of their choice. Before and during the stress exposure, we measured participants' state anxiety through self-reports and also physiologically with heart rate (HR) using a chest belt and a smartwatch (Suunto Ambit3 Run, Suunto, Finland). Upon arrival at the lab, participants were given 10 min to warm up and were then equipped with the chest belt. Thereafter, they moved to another room (i.e., the performance venue), where they first sat quietly for a 90-s baseline and then performed the mental arithmetic task in front of a jury and a video camera. The jury consisted of two persons who were not introduced to the participants and who were instructed to behave neutrally. The task was to sequentially subtract the number 13 (pretest) or 17 (posttest) from 1,022 and verbally report the answers aloud for 3 min. If they made a mistake, they were made aware of it and asked to start over from 1,022. Immediately after the arithmetic task, participants filled in the Mental Readiness Form-3 (MRF-3; Krane, 1994) to self-report their state anxiety. In particular, they set marks for their present feeling on three separate 100-mm lines, which were anchored between *calm* and *worried* for cognitive anxiety, *relaxed* and *tense* for somatic anxiety, and *confident* and *not confident* for self-confidence. The measured length between the left end of the line and the participant's mark was calculated as a score out of 100, with higher scores indicating higher anxiety. Participants then stood still for another 90 s before performing audition excerpts of their choice, which lasted on average 4.0 min (*SD* = 1.2). Detailed information on their audition tasks can be found in Supplementary Table S1. To measure how quickly their HR would return to baseline, they then remained still for another 90 s. To minimize the effect of merely improving newly learned excerpts over the following weeks, they had been asked to have prepared them in the way they would for an audition. Finally, after completing the audition excerpts, participants were interviewed about their performance experience. We developed an interview guide similar to Mesagno et al. (2008): a semi-structured approach with pilot-tested, open-ended questions (i.e., prefaced by how? what? in what way?). The interviews explored participants' performance experiences, their focus, emotions, and mental strategies, and their evaluation of how they performed. The pretest interviews took 22–44 min (*M* = 29.4, *SD* = 6.6).

The *posttest* was identical to the pretest, but preceded by the questionnaires from the selection phase a second time. It was required that participants perform the same audition excerpts as in the pretest. The semi-structured posttest interview included questions referring to each participant's interventions in addition to the questions also asked in the pretest. They closed with questions about other potential influences during the intervention time, what participants had learned during the study, and what they took away from the coaching. Posttest interviews lasted 29–63 min (*M* = 44.8, *SD* = 11.3). After the interview, participants were paid and thanked.

Upon the completion of data collection, interviews and coaching sessions were transcribed verbatim using the transcription software f4transkript (audiotranskription, Germany). The overall performance quality of all anonymized video recordings was evaluated individually on a scale from 1 (*bad*) to 10 (*excellent*) by nine experts, who were renowned professionals, professors, and/or judges for auditions and competitions in the respective domains. They were instructed to do so in the same way they would for a professional audition procedure. Given the variety of domains, instruments, and genres, as well as the extensive amount of video material per participant, they only rated the respective participant for whose field they were indeed experts, so there was one expert rating per participant.

### 2.4. Intervention

During the 10-week *intervention* phase, participants received five bi-weekly individual coaching sessions, with an average duration of 54.0 min (*SD* = 4.9), from the first author who is a psychologist and certified psychological coach. To ensure the personal relevance of the interventions, participants were encouraged to express their own goals for the whole intervention period as well as each coaching session. Interventions were then tailored to each participant's specific problems of performing under pressure by accommodating their own goals and requests during each session. Six participants wanted to increase their self-confidence, four asked for strategies for mental performance preparation and self-help, and two also wanted to enjoy performing and get immersed in the moment.

Every session was somewhat unique, but in the following, we describe certain recurring elements. Some typical questions referred to the participant's hopes for ideal outcomes of the entire 10-week period (first session) as well as for each session at the beginning, how the participant had been doing the 2 weeks before (sessions 2–4 in particular), how the coach could support their concern(s), and which specific steps they would take in the following weeks (sessions 1–4). Session 5 was introduced as an opportunity to reflect on the past sessions, on aspects that had been helpful, and on how participants envisioned to continue in future (e.g., among the closing questions was “what would you like to tell your future self?”). When participants had expressed a certain goal or need, they received psychoeducation about potentially matching psychological strategies. The chosen strategy was then tried out together, sometimes written down by the participant, or modified/adapted according to their experiences either during the session or in the field.

The tailored choking interventions included PPRs, goal-setting, and acclimatization training (see Table 2). To help participants with the implementation of these interventions, they were provided with instructions in imagery, self-talk (including reappraisal cues), and techniques for relaxation and concentration, such as centering (Greene, 2002), left-hand contractions (Beckmann et al., 2013), and deep breathing. In order to match participants' individual goals and needs, some were additionally instructed in Progressive Muscle Relaxation and Autogenic Training. Participants were requested to test the practical application of their respective interventions in simulated or actual performances. Simulated performance scenarios were created by the participants themselves, whereas actual ones included professional auditions and graded performances at university. Participants were asked to submit weekly video recordings of these performances.

**Table 2:** Tailored interventions for each participant.

		**Intervention**					
	**Goals**	**PPR**	**Imagery**	**Acclima-tization**	**Relax-ation**	**Goal-setting**	**Self-talk**
Anne	Improve self-confidence, stage presence, and control	✓	✓	✓			
Bianca	Improve self-confidence	✓	(✓)	✓	✓	✓	✓
Coco	Improve mental performance preparation, learn strategies and techniques	✓	✓	✓	✓		
Julia	Show ability on stage, play from memory without blackout, enjoy performing	(✓)	✓		✓		✓
Lucy	Improve self-confidence, immerse in the moment, let go	✓	✓				✓
Mia	Understand own behavior during performance, improve stage presence	✓	✓	✓	(✓)		✓
Tom	Learn strategies for self-help	✓	✓	✓	✓		
Vivi	Improve self-confidence, show ability on stage	✓			✓	✓	✓
Zoe	Improve self-confidence	(✓)	(✓)			✓	

(✓), intervention was implemented together; (✓), technique was being discussed, but either already learned before the study or not implemented during the intervention phase.

### 2.5. Quantitative analysis

Questionnaire data, quantitative expert evaluations, and HR measurements were analyzed using SPSS 27.0 (IBM Corp.; Armonk, NY, United States). Questionnaire scores were computed by averaging the responses across respective items. In addition, we determined percentile ranks for each participant in relation to the norm. To analyze HR during pretest and posttest, we applied the formula from Pruessner et al. (2003) to calculate the “area under the individual response curve with respect to the ground” (AUC_G_) for each participant. To complement our qualitative and descriptive data, we used paired-sample *t*-tests, with a level of significance at *p* < 0.05 (one-tailed).

### 2.6. Qualitative analysis

We analyzed the transcripts of interviews and coaching sessions using reflexive thematic analysis (Braun and Clarke, 2021) with Quirkos (Quirkos Limited, United Kingdom). The first author, who also collected the data, kept a reflexive journal throughout data collection and analyses and engaged in extensive reading and re-reading of transcripts to enhance familiarity with the data. She began the coding process focused on the research aims, while also noting passages potentially relevant to illuminating the underlying mechanisms of the interventions. Arising issues and questions were frequently discussed with the second author. Having re-read and refined initial codes, we developed preliminary themes together, including descriptions, subthemes, and exemplary quotes. The third author served as a critical friend to challenge and discuss these initial themes and encourage alternative understandings (Smith and McGannon, 2018).

## 3. Results

As we consider our quantitative and qualitative results to be of equal importance, we present them in an interleaved fashion, structured according to the different outcomes we investigated. Quantitative results are displayed in Table 3, and the themes from the qualitative analysis are displayed in Table 4. Quotes from participants to describe the themes are provided without corrections in English or as translations from German.

**Table 3:** Results of paired-sample *t*-tests with effect sizes (Cohen's *d*_z_).

**Variable**	***M*** **(*****SE*****)**		***t*** **-Test**		
	**Pre-test**	**Post-test**	***t*** **(8)**	***p***	*d*_z_
**Traits**				
Trait performance anxiety	4.00 (0.39)	2.92 (0.27)	−2.88	**0.010**	0.96
Fear of neg. evaluation	4.15 (0.13)	3.38 (0.26)	−3.81	**0.003**	1.28
Self-efficacy	2.81 (0.18)	3.23 (0.14)	3.10	**0.008**	1.02
**States**				
Cognitive anxiety	55.44 (4.67)	24.44 (5.15)	−5.72	**0.001**	1.91
Somatic anxiety	65.11 (6.05)	33.22 (8.06)	−3.26	**0.006**	1.09
Confidence	50.67 (8.02)	67.33 (7.37)	4.15	**0.002**	1.38
Heart rate (AUC_g_)	7,761.05 (570.17)	7,385.59 (505.92)	−1.38	0.102	0.46
**Performance**				
Performance quality	6.64 (0.54)	8.11 (0.43)	2.80	**0.013**	0.93

AUC_g_, area under the curve with respect to the ground. Significant *p*-values are marked in bold.

**Table 4:** Overview of themes and subthemes with examples.

**Theme**	**Subtheme**	**Example**
Managing nervousness and feeling relaxed	Experiencing nervousness	“I was so scared that I would like not gonna make it” (Zoe)
	Struggles due to performance circumstances	“The hall is extremely annoying to play in” (Vivi)
	Feeling more relaxed and calm	“Actually I felt really calm” (Mia)
Becoming more self-confident and more proud	Confidence in one's ability to perform and to deal with unexpected situations	“Generally I simply have a bit more confidence now that I do indeed play well in any case” (Bianca)
	Being more courageous	“It was fun to try it out. Rather than like, ‘oh gosh, I was anxious about it”' (Coco)
	Less concern about negative evaluation	“I care less about what people think about me” (Tom)
	Stronger sense of artistic identity and pride	“I was really proud and I felt like (...) ‘yes, I was born to do this!”' (Zoe)
Satisfaction with artistic and mental performance	Performance highlights	“A highlight was now actually the intermediate exam” (Julia)
	Performance went well	“It went well, like it's just, I didn't expect to” (Coco)
	Own expectations were met	“I implemented exactly what I had aimed for” (Lucy)
	Recognition of sub-par aspects	“The intonation was unfortunately not good, also because of the weather and because of me” (Vivi)
	Other's feedback and perceptions	“Every teacher told me beautiful things. I got really good feedback” (Zoe)
Feeling good and enjoying performing	Feeling comfortable	“I have felt rather secure most of the time” (Tom)
	Having fun	”It was really fun then in the moment” (Lucy)
	Flow experience	“Now I think I was more in the flow” (Mia)
General positive effects of the intervention		“I think these techniques are always helpful, not just for the performance, but also generally when one is stressed or somehow, yeah, confronted with a difficult situation or when one does not know how to deal with something” (Anne)

### 3.1. Experiencing and managing performance anxiety

**Figure 2:**
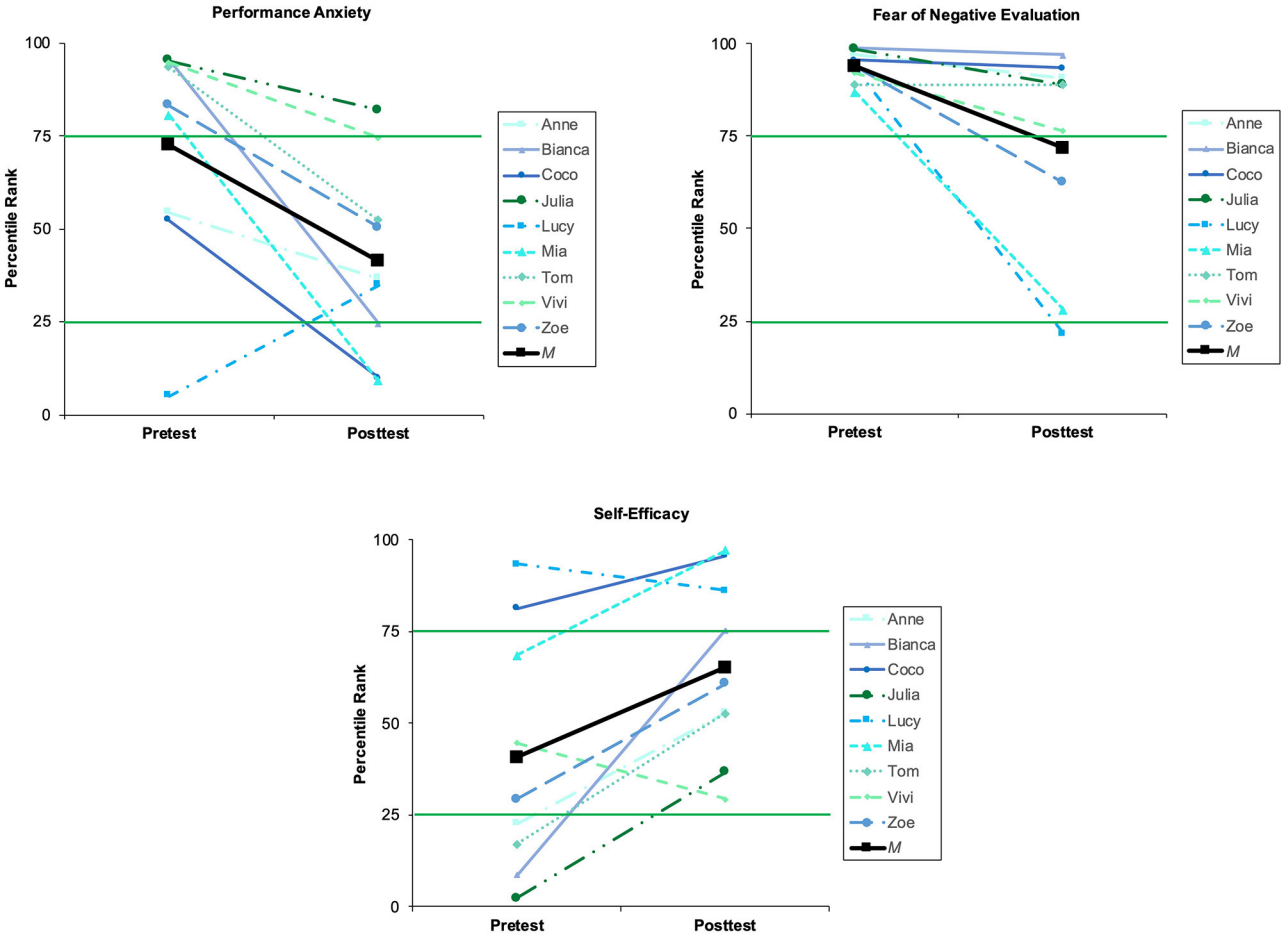
Percentile ranks for trait performance anxiety, fear of negative evaluation, and self-efficacy in pre- and post-tests.

Both trait performance anxiety and FNE appeared to significantly decline from pre- to post-intervention (Figure 2). One participant, Lucy, showed an increase in trait performance anxiety, which might be explained by a rather low percentile rank in the K-MPAI she had in the pretest, presumably because she underestimated how anxious she actually is. As she expressed regarding an important performance: “...and so suddenly ‘bam', the whole nervousness, which I haven't known like this in a while, was there.” Participants never used the term anxiety, but referred to nervousness or stress instead, therefore we consider them as synonyms. Despite the overall decline in anxiety, *experiencing nervousness* before performances was still an important theme for some participants: “I thought that I had left this behind, but there it was again” (Tom); “I was so stressed before the stage” (Zoe). They described becoming anxious often because they felt like they had to prove their artistic ability and show everything they had prepared: “About 2 seconds before I went on stage, there was the thought ‘You're only allowed to show this once, you may never do it again, you now have to show everything you thought, and ideas, and just have fun now, because you can never repeat this, it's now the only and last time'. And your friends are watching and the jury is watching” (Lucy).

The intensity of the anxiety experience was amplified by the fact that participants had not been on stage for months because of the COVID-19 pandemic. Their nervousness was, therefore, also elicited by *struggles due to performance circumstances*, such as unexpected situations or adverse conditions. In particular, there was a feeling of not being able to communicate with the audience when face masks had to be worn during the performance. Other struggles that inhibited communication were caused by bad acoustics or having to play behind a wall in an audition. Participants further talked about being overwhelmed by the sudden increase in performances after lockdown and a subsequent lack of preparation and time pressure: “the problem was that I had had too little preparation. (…) It was just everything within a very short time. It was simply too much” (Vivi).

**Figure 3:**
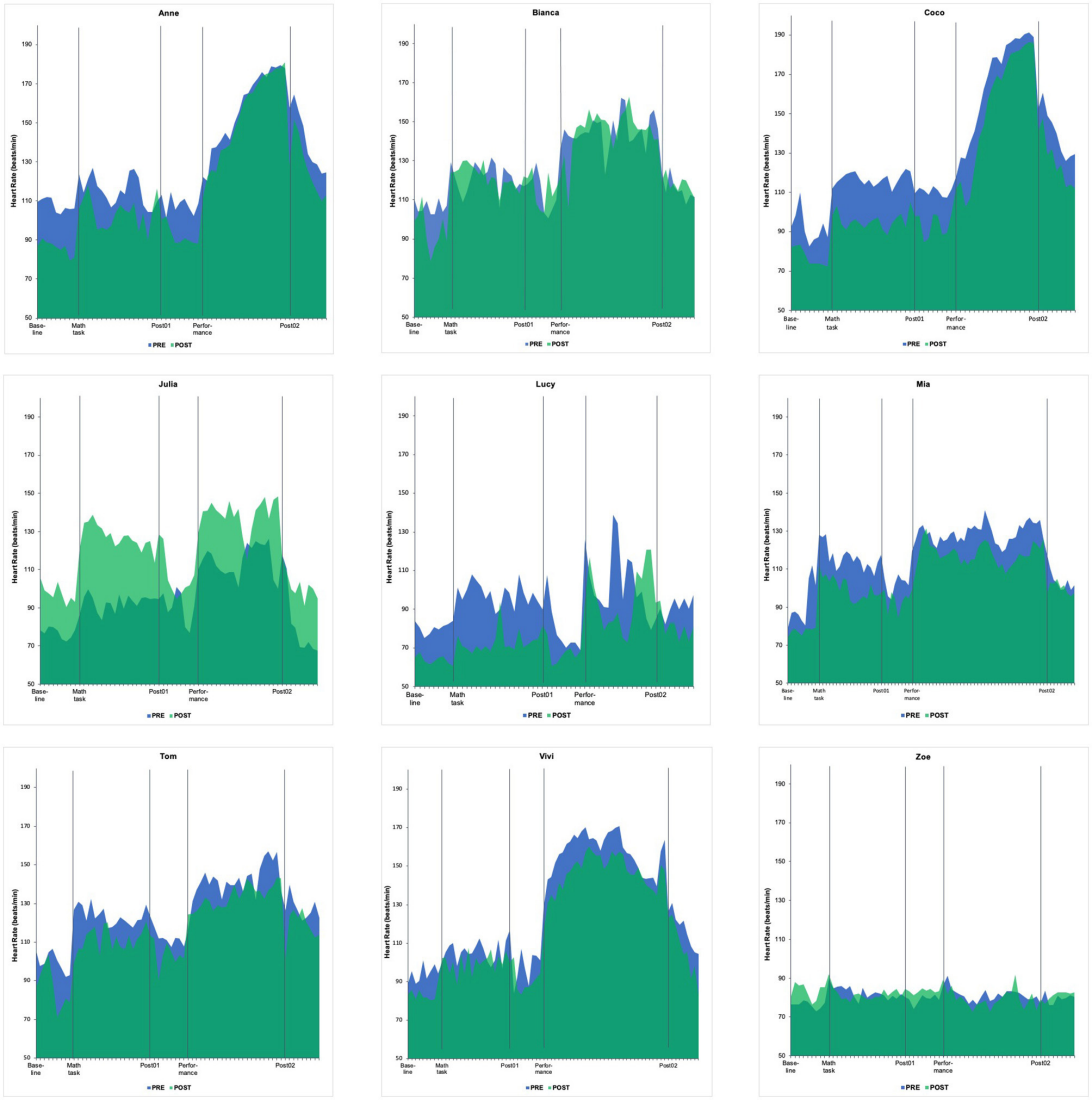
Heart rate in pre- and post-tests.

Notably, both quantitative and qualitative data indicated that participants learned to better manage their anxiety. They generally reported lower state anxiety before the posttest performance, with significantly lower values in both cognitive anxiety and somatic anxiety in the MRF-3 compared to the pre-test (Table 3). HR similarly decreased from pre- to post-test (Figure 3), but not significantly so. Furthermore, an important theme was gradually *feeling more relaxed and calm* during performance after learning, and experimenting with, different strategies during the intervention period: “and then I actually could calm me down. And that was a really cool feeling” (Mia). Other participants similarly described managing their nervousness effectively. Lucy in particular was able to learn from her dissatisfaction with one performance, improving her anxiety management and performance during a subsequent audition:

I have now noticed what it is when I am too much. I have noticed what it is when I am too little (. …) ‘how do I achieve this balance?' And I somehow then managed that in the audition there and I was totally good, it was really fun.

The effect of applying the interventions was also perceived as an absence of physical symptoms, such as shaking, palpitations, or being cold: “I didn't have so many palpitations, so I mean with me it is often like that, mainly due to excitement or stress, that I really, yeah, that it immediately manifests itself physically, but this time it did not” (Anne).

Most participants exhibited a lower HR in the posttest, yet the HR measurement itself also led to discomfort: “what really bothered me is that the thing here [the chest belt] actually made me/ like I could feel my heartbeat all the time” (Mia); “this is kind of also stressful to see someone measuring your heart rate” (Zoe). Julia's HR even increased by 26% in the posttest. However, she evaluated this positively: “I arrived quite tired because it was a hard week (…) And I was actually rather glad that a bit of nervousness came, as one becomes more awake then”. This illustrates that heightened physiological arousal before a performance does not necessarily need to be debilitative. In the same vein, Julia said about her successful recital a few weeks before the posttest: “I was monstrously nervous, of course. But then it wasn't a problem.” Vivi even remarked that she needed a certain amount of nervousness for optimal performance: “the reason for some mistakes is that I was not alert enough (.…) The concentration is not quite there (.…) When I'm nervous, I am always a tad more precise”. Taken together, participants' performance anxiety decreased in the posttest, but they also acknowledged that they felt nervous in certain situations. This was sometimes facilitative, but often had to be regulated via an anxiety management strategy.

### 3.2. Strengthened self-confidence and artistic identity

Overall, participants appeared to show both significantly increased general self-efficacy after the intervention (Figure 2) and significantly higher confidence before the posttest performance (Table 3). Lucy and Vivi, however, reported slightly decreased self-efficacy in the GSE in the posttest. Interestingly, this did not correspond with their interviews. Lucy dropped from an already high percentile rank of 93 in the pretest to 86 in the posttest, yet in her interview, she said about the coaching: “it has really greatly strengthened my self-confidence.” Similarly, Vivi stated in contrast to her questionnaire: “I am now in a phase in which I am getting much more self-confident.”

Our thematic analysis revealed that *confidence in one's ability* was expressed as trust that one *can* indeed play and perform, and having this trust allowed participants to let go more and not control too much: “I can play, because in that moment I could rely on it going well (.…) there I could listen relatively well. Yeah and that was very beautiful” (Julia). This confidence was extended to having strategies for managing anxiety and uncertainty: “and so generally I simply have a bit more confidence now that I do indeed play well in any case, but can also learn how to deal with my, yeah, nervousness” (Bianca); “that I can optimize my preparation (…), but also be ready to adjust” (Coco). Knowing such strategies gave participants a sense of security: “and there I was so certain and secure that nothing could faze me in that moment, nothing disturbed me” (Lucy); “I believe it just gives me like a general feeling of a bit of a greater security” (Tom).

Having trust and security, participants also described themselves as *being more courageous*, e.g., when improvising, taking risks on stage, or trying new things: “I was totally free (....) I knew nothing can happen now” (Lucy). Some also considered it courageous to show themselves on social media: “I already posted the videos from [prestigious performance venue] (...) I have never ever posted a video or a photo of myself on the stage before that. Never. (…) That's a big step” (Zoe). With their strengthened self-confidence, they noticed *less concern about negative evaluation*: “I'm actually not afraid of other musicians anymore” (Mia); “I didn't think much of like ‘how do I look?' and what people think” (Zoe). This is also underlined by the questionnaire results on reduced FNE described above.

Finally, enhanced self-confidence became visible in expressions of a *stronger sense of artistic identity and pride*. Being proud comprised all aspects of being an artist: “that I can indeed be proud of myself and that actually everything is alright the way it is, how I play and all” (Bianca); “I was really proud and I felt like ‘yes I was born to do this, like this is me, this is what I should do”' (Zoe). Especially after experiencing several lockdowns, being or becoming aware of one's artistic identity was quite powerful and meant to be fully connected to what one is doing: “it's like safe, it's kind of indestructible now, it's me and the violin” (Mia). Zoe elaborated that she was “embracing like who I am. And I was kind of lost, maybe because of lockdown (…) I'm a musician. (...) I'm like very proud of what I'm doing, what I'm going to do and I can see the future.”

### 3.3. Positive feelings during performance and satisfaction with performance quality

**Figure 4:**
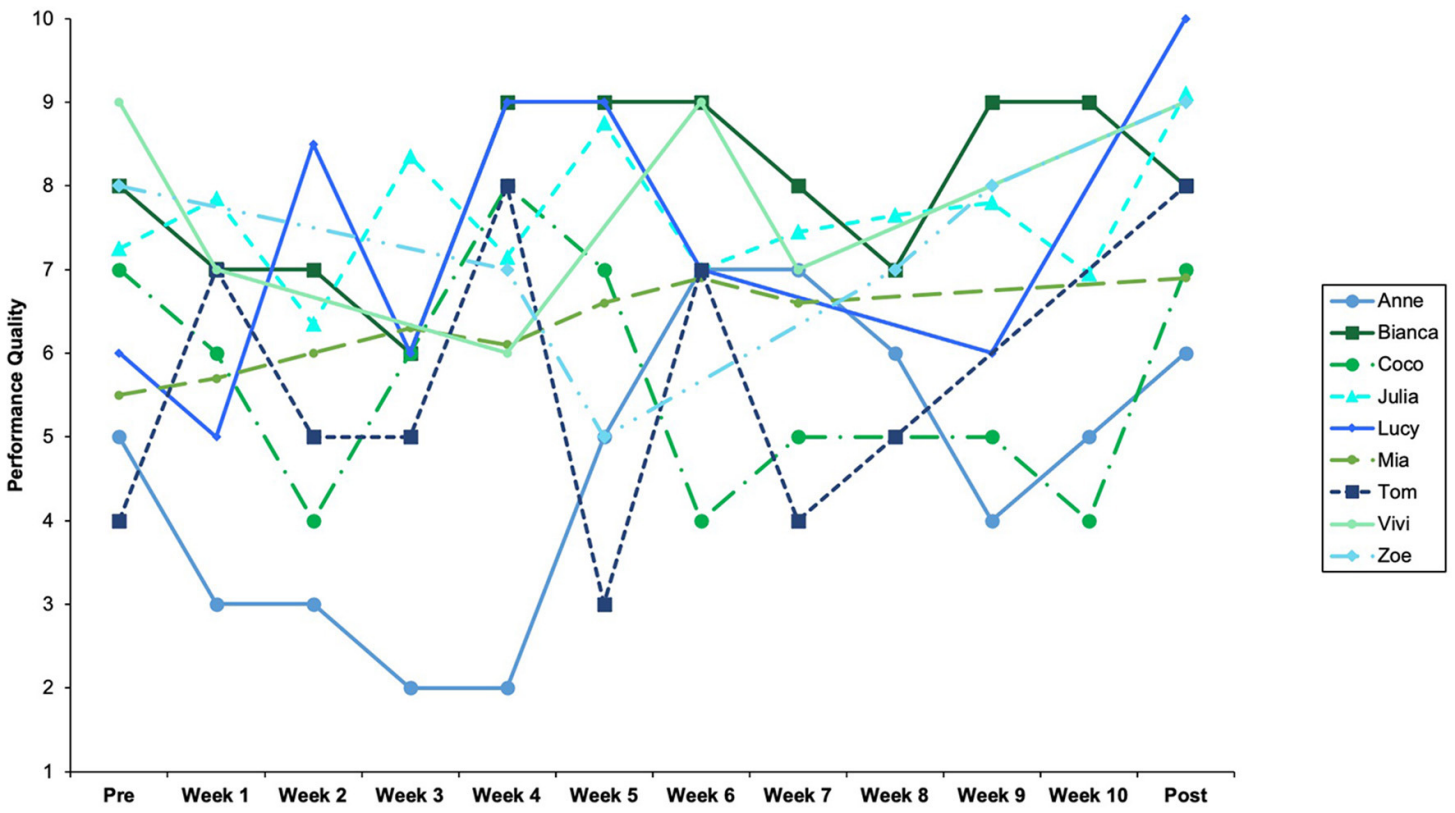
Expert performance evaluations.

Performance quality appeared significantly higher in the posttest compared to pretest performances (Table 3). The expert ratings for pre- and post-tests and weekly performance videos are displayed in Figure 4. It should be noted that when comparing pre- and post-tests on an individual level, performance quality either increased or remained stable. For the intervention period, however, performance quality fluctuated strongly for all participants except Mia, whose performance varied slightly, but otherwise slightly increased. For some participants, *performance highlights* occurred both during the intervention phase and in the posttest: “for me a highlight was now actually the intermediate exam” (Julia); “whoa, I found it fantastic today (laughs) (.…) and I was completely inside the character” (Lucy). Others mentioned that the *performance went well*, often despite adverse circumstances: “it was really crazy. But somehow it went well, like it's just, I didn't expect to” (Coco). Participants further expressed their satisfaction in describing how their *own expectations were met* by their positive performance experiences: “all what I had wished for, what I want to achieve with the monolog and after the training, exactly that I have accomplished today. (…) I'm one hundred percent satisfied” (Lucy); “so this was just pretty close to that ideal, what I just have there, or it was exactly that, how I actually wish it to be” (Tom).

However, not all performances were completely satisfying, as indicated by participants' *recognition of sub-par aspects*. They gave different reasons for why they performed less than desired and also alluded to the importance of context:

Today was a chaotic day for me. And like physically I don't feel so good (…) just internally focused on the music and on the steps and trying not to focus on the pain and on the audience and additional stress. (…) And I felt really in it. I mean it wasn't perfect, there was some little things that could have been better, I think. (Coco)

Other aspects included intonation and technical issues, taking more time or preparing particular moments better and lacking artistic excellence: “it was not quite clean, that is of course a matter of practice, that the brilliance is a bit lower and it also does not sound so lively” (Julia). Participants justified sub-par performance with a lack of preparation or difficult circumstances or evaluated themselves worse when they were comparing the performance to previous, better ones. Going beyond artistic performance quality, participants also talked about satisfaction with progress in the psychological dimension of their performance: “and the performance like mentally, I am actually also really satisfied. I have actually also managed that well” (Bianca); “so it is now more, perhaps one can then let one's body (…), let one's skills just do, one does not worry so much anymore” (Anne).

An important part of their performance evaluations concerned *feedback from others*, such as professors or members of the audience or jury. When participants were satisfied with their performance, positive feedback was perceived as encouraging or rewarding their effort: “when one gets praise from one's professors, also from non-accordionists, that/ and one sees, ‘ah yes, it was then somehow worth it'. Crazy often for months, I would run into a wall when playing from memory. But then it somehow did work out” (Julia). Discrepancy between the inner and outer perspective was perceived as interesting or illuminating: “that the self-evaluation after playing sometimes diverges quite substantially from what one would say if one had watched oneself from the audience's perspective” (Tom). Others' feedback was described as frustrating when it differed from one's self-evaluation in a negative, but also in a positive way, especially when not seen as constructive. In terms of the performance experience itself, one's own perception might actually be more important, as expressed by Vivi: “that is then actually horrible for me to play, when I myself do not [perceive] it as beautiful”.

With the theme *feeling good and enjoying performing*, we extend the idea that the artists' own experience may be crucial. We identified a pattern of emphasizing one's feelings against technical or artistic evaluations of one's performance. In particular, Vivi stated: “I somehow find it more important how I'm doing (…) when I feel good, then of course the audience feels good, too”. On the one hand, *feeling comfortable* was explicitly related to a higher layer of experience: “I could listen relatively well to what I wanted to do and could divert a bit from this technical aspect (…) it is mainly that I now really had the feeling (…) that I can just make music” (Julia). On the other hand, feeling comfortable during the posttest was also described with different facets. Sometimes it was attributed to being more familiar with the situation: “I think I generally felt more comfortable (.…) also because I of course have already experienced the situation once, that naturally makes a difference” (Anne). Simultaneously to providing comfort, this familiarity also increased the pressure on some participants. Being aware of the comparability raised the expectation of performing substantially better than in the pretest: “I have put a bit more pressure on myself today, because I naturally, yeah, somehow just also wanted to show an improvement” (Bianca). Finally, when having to communicate dramatic emotions, feeling comfortable might even be counterproductive: “I was feeling very comfortable and I actually thought ‘Oh dear, how should I play this monolog now? I'm in way too good a mood”' (Lucy).

*Having fun* was described as a particular way of enjoyment during the performance and related to a positive energy that was shared with one's ensemble or audience:

At my concert it was truly like that, that I had really a lot of fun to play with the people (…) that also came like, as feedback from my fellow musicians, that they somehow also had fun and that it was a cool ambiance and energy. (Tom)

Fun and enjoyment were mentioned as distinct features of the posttest and were connected to being in the moment:

At the first one I was like kind of ‘okay let's sing it and let it be over. Let this moment to be over.' And today I was kind of more enjoying it. Like I could sing more, like I hold the last note. (Zoe)

Ultimately, enjoying the performance and being in the moment was connected to *flow experiences*, characterized by participants as being “simply completely in the zone” (Tom), “not thinking about doing it a certain way” (Coco), and “being rather than showing or doing” (Anne). For Lucy, this constituted her main goal for the coaching, and at the end, she reflected on its meaning:

For me, everything culminates in being in the moment. And through the exercises, I've accomplished that now, so I would say, I have/when I have managed to be in the moment, then I have indeed also achieved something in the other steps.

### 3.4. Broader effects of the intervention

As a final theme, we identified *general positive effects of the intervention* that went beyond the effects outlined above. Enhanced motivation was explicitly mentioned as something participants took away from the coaching. For example, Zoe said:

I get a lot of motivation. Like, not like ‘okay let's get out of the bed and practice', not that. I mean I get motivation of singing again in shower, every moment of the day, and listening to music actually more, and enjoying it. Enjoying every noise that I'm hearing. So that was really nice. Thank you for that.

Participants were mostly content when looking back at the coaching process and their initial goals, but sometimes they also changed the goals themselves, which opened up new perspectives: “not to look for perfection or stability in every area, that ‘now I'm prepared for everything', but also if I'm not prepared to something, that I can deal with it” (Coco). Using intervention strategies was described as effective and uplifting for how one talks to oneself, not just regarding performance, but also in everyday life: “I can deal better with agitation in general, or with the/ maybe also with thoughts or attitudes toward myself that are not helpful, but are in the way or rather hold back” (Anne). Indeed, being aware of one's ability to manage one's thoughts, feelings, and reactions, also through what was learned during the study, appeared as the main asset and take-home message:

That I really feel like this, that I went from monochrome to full colors (.…) Like in almost every way when it comes to performing, like how my body reacts, how my mind is set up, how do I like interact with others, and this big calmness what is the current topic of mind, that's also coming, and that also changed my everyday. (Mia)

## 4. Discussion

In this study, we sought to illuminate the effects of tailored interventions on choking-susceptible performing artists' performance quality, performance anxiety, fear of negative evaluation (FNE), and self-efficacy with a mixed-methods approach. Amidst variation both between and within individuals, both qualitative and quantitative data indicate a positive impact. This includes higher performance quality according to experts as well as participants, enhanced self-efficacy, and lower performance anxiety and FNE in the posttest. Thematic analysis of interviews and coaching sessions revealed that this progress was not always characterized by linear improvement: instead, some experienced struggles with intense nervousness and subsequent performance decrements, but also performance highlights earlier in the intervention phase that made the posttest seem less remarkable in comparison. The positive effects of the intervention were perceived not only in relation to cognitive, emotional, and behavioral aspects of performance, but also extended to participants' everyday lives.

Questionnaire and interview data appeared to show that participants had lower trait and state performance anxiety after the intervention period. During the intervention phase, however, some performances elicited strong anxiety. Participants were either able to manage their anxiety so that it did not negatively impact performance quality, or to learn from anxiety-provoking situations and thereby improve subsequent performances. Familiarity with the setting in the posttest created a more comfortable feeling, but at the same time also increased the pressure to perform even better than in the pretest. The relationship between perceived pressure of the situation and performance anxiety thus appears to be individual and context-dependent. Importantly, participants' interpretation of their anxiety symptoms, but also the controllability of these symptoms through psychological strategies, may be more important than their intensity.

The relevance of interpretation and controllability has been pointed out in previous studies with actors (Goodman and Kaufman, 2014), dancers (Walker and Nordin-Bates, 2010), and musicians (Clark and Williamon, 2011). These studies link feeling in control of one's anxiety to self-confidence and self-efficacy, but with different directions: feeling in control may be the result of raised self-confidence and subsequently foster facilitative interpretations of anxiety (Walker and Nordin-Bates, 2010), or feeling more in control of debilitating aspects of anxiety may lead to increases in self-efficacy (Clark and Williamon, 2011). Notably, the intervention by Clark and Williamon did not reduce musicians' performance anxiety, but it did enhance their self-efficacy. Participants in our study depicted anxiety management strategies as strengthening their self-confidence, as well as their self-efficacy for both dealing with their anxiety and performing well. In contrast, the reduction of FNE, as shown by both quantitative and qualitative data, appeared to have been grounded in participants' enhanced self-confidence and pride. Given the potential role of FNE in the relationship between self-presentation and choking (Mesagno et al., 2012), lowered FNE through increased self-confidence may be a particularly noteworthy outcome.

The enhancement of self-confidence became especially apparent in participants' descriptions of daring to do things they had not done before, and of being more aware and proud of being an artist. Exhibiting stronger identification and connection with one's art form after taking part in the study should also be seen in the context of the COVID-19 pandemic, during which performing artists were confronted with existential challenges (Spiro et al., 2021). Our study was conducted at a time when it became gradually possible again to perform, giving participants the opportunity to receive appreciation from an audience and reconnect with their profession and artistic identity.

Feedback from others was an important part of how participants saw their performance. Whereas unfounded feedback may cause anxiety, positive and constructive feedback may enhance self-confidence (Walker and Nordin-Bates, 2010). From the standpoint of self-efficacy theory, participants' successful performances may have represented mastery experiences that supported their self-efficacy for performing and subsequently facilitated satisfying performance experiences in the posttest (Bandura, 1997). Whereas previous research in music found that self-efficacy predicts performance quality (e.g., Ritchie and Williamon, 2012), their causal and bi-directional relationships have to be investigated more thoroughly.

For reasons of article length, it is not possible to discuss each participant's individual goals and interventions. However, some insights deserve mention. Remarkably, none of the participants set the goal of explicitly reducing anxiety, and only two of them wished for improved performance in the sense of being able to show on stage what they had prepared. Therefore, in addition to the choking interventions that have so far been beneficially applied by athletes, the tailored interventions in this study also included additional relaxation techniques to accommodate participants' requests for methods to help them sleep better or deal with excessive muscle tension. Imagery and self-talk also became relevant to improving performance from memory, enhancing video recordings for auditions, or facilitating emotional transitions between different parts of a show. Some participants also expressed the need for managing their post-performance emotions and arousal. These are examples of immediate and surrounding aspects of performance under pressure that appear to be of particular significance to choking-susceptible performing artists and could be helpful to inform future studies and interventions. The variety of requests and preferences also attest to the truly multifaceted nature of performance anxiety and the importance of taking an individualized approach.

Participants' accounts of enjoying performing and the general positive effects of the intervention also point toward a promising two-pronged approach for future interventions, adding the perspective of positive psychology and enhancement of flow to anxiety management. Indeed, such an intervention by Cohen and Bodner (2019) resulted in better performance and lower performance anxiety. Whereas they did not report changes in global or dispositional flow after the intervention, the authors argued that changes in flow state as a short-term experience after a performance may be more relevant and readily observable in relation to PST. Similarly, descriptions of flow states by our participants were connected to specific performance experiences. Therefore, participants' emphasis on being in the moment and the ability to let go, but maybe also accept certain thoughts and emotions, points further toward the relevance of ACT and ACC as a possible, alternative or complementary, way of intervening with choking-susceptible performing artists (Juncos and de Paiva e Pona, 2022).

### 4.1. Applied recommendations

Tailoring interventions to individual needs and goals appears to be a promising avenue for supporting performing artists who are strongly affected by their performance anxiety. Learning strategies for performance under pressure seemed to be relevant not only before, but also after performances, as well as for general stress management and wellbeing in their artistic everyday life. The most important recommendation is thus to let artists self-determine the implementation process of their tailored intervention. Occasionally, intervention strategies were discussed in the coaching sessions without subsequently being applied by participants, mainly because there was not enough time or even perceived necessity to do so. Allowing enough time for implementation and assisting in either finding situations to test out strategies or actively creating them, such as with acclimatization training, is therefore crucial for putting tailored interventions into practice. Even when performing artists feel restricted by their anxiety and are thus choking-susceptible, the focus of intervention may have to be directed more toward finding flow and being in the moment, especially when their own perceptions and emotional experience of the performance are more relevant to them than audience evaluations.

### 4.2. Strengths, limitations, and future research

This study is the first to investigate tailored interventions with choking-susceptible performing artists. The mixed-methods approach and the wealth of data collected for this study are its major strengths. That the first author, a psychologist, certified psychological coach, and trained violinist, both conducted coaching and interviews and analyzed the data, can be seen as a strength as well as a limitation. With her background in music and affinity to dance and acting, she was able to establish rapport with the participants and empathize with performance-related challenges, which may be vital for implementing sports-based interventions with performing artists (Pecen et al., 2016). Furthermore, she could use personal insights and profound knowledge of the data as assets for reflexive thematic analysis (Braun and Clarke, 2021). At the same time, her involvement likely shaped how participants evaluated the interventions' outcomes, and the way she approached the analysis.

The individual, person-based tailoring approach may provide benefits for performing artists but also makes direct replication impossible. We conceptualized this study as a collective case study in order to explore such a tailored approach and its effects. The lack of replicability is a weakness that we believe is somewhat inherent in our approach. A detailed analysis of how the first author as coach tailored interventions to participants' goals or expressed needs was not included in this article for reasons of article length, but might be an interesting subject of future research. Furthermore, we would like to differentiate our approach (i.e., having a psychologist deliver tailored interventions within an individual coaching setting) from PST and ACC programs delivered in group settings by performing arts educators without specialized psychological training (Gill, 2020; Shaw et al., 2020; Mahony et al., 2022). Future studies might thus be focused on whether or how performing arts educators can be trained to tailor such interventions specifically to performing artists' individual needs.

Because not all the originally selected artists were able to participate, and because the distribution in the population invited to participate was uneven between music, dance, and acting, the three domains were not equally represented. In addition, some participants did not meet all three quantitative selection criteria. Future studies may benefit from recruiting choking-susceptible participants from a larger pool of performing artists. That some of those originally chosen withdrew their participation was also due to the unique situation during recruitment: performances were finally possible again after over a year with several lockdowns. This situation should be considered when looking at the results: a certain enthusiasm, but also overexcitement about being on stage again may have been due to the contrast to previous restrictions.

Furthermore, the sample would be too small for a purely quantitative study. With the data of nine persons, we had 70% power to observe a large effect of *d*_z_ = 0.8, indicating that larger samples should be used in future studies that mainly focus on quantitative effects. In terms of performance quality, budget restrictions and the extensive amount of video material meant that each participant was only evaluated by one expert in their respective field. Future studies may include several raters per performance to ensure higher reliability of performance evaluations.

There is recent evidence that repeated performance exposure can significantly reduce HR and anxiety-related non-artistic performance errors (Candia et al., 2023). Not having included control cases might be seen as a limitation for the quantitative analysis, yet we could not conceive of an adequate control condition for our extensive design. We, therefore, emphasize that the outcomes may not be exclusively attributable to our intervention. In addition, future studies may take into account context-specific factors that could influence performing artists' perceptions of their environment (Miller and Chesky, 2004).

Finally, causal effects may be difficult to disentangle, even for the participants themselves. Some participants noted that it was hard to understand which specific effect could be attributed to the intervention: “always difficult to find out for oneself, what is why and how, because yeah one does not really have a suitable comparison” (Tom). Therefore, the causal effects of the interventions cannot always be implied. Future research might investigate possible underlying causes and mechanisms along with artists' abilities in applying psychological skills, and not just focus on the outcomes of an intervention.

## 5. Conclusion

This study strengthens the bridges between different performing arts as well as between performing arts and sport psychology. Our data demonstrate that tailored interventions inspired by sport psychology can have a positive impact on choking-susceptible performing artists' anxiety, self-efficacy, performance quality, and even their everyday lives. In our view, the qualitative data particularly emphasize the importance of individual context. Whether an intervention can be considered beneficial also depends on the perspective taken: is it more relevant to improve the artist's or the audience's satisfaction with the artistic quality of the performance, or perhaps just artists' own emotional experiences while performing? Future research should thus be extended to the investigation of long-term effects and underlying mechanisms of tailored interventions, potentially with a stronger focus on how the interventions are being implemented and what role the relationship during coaching plays.

## Data availability statement

The raw data supporting the conclusions of this article will be made available by the authors, without undue reservation.

## Ethics statement

The studies involving human participants were reviewed and approved by Institutional Review Board of the Department of Work, Economy, and Social Psychology, Faculty of Psychology, University of Vienna. The patients/participants provided their written informed consent to participate in this study.

## Author contributions

VL and PG conceptualized, designed, and conducted the study. VL implemented the interventions and wrote the first draft. SN-B conceptualized the qualitative analysis. PG and SN-B wrote sections of the manuscript. All authors contributed to data analyses and manuscript revision, read, and approved the submitted version.

## Footnotes

^1^The Trier Social Stress Test (TSST) is the most widely used and well-validated laboratory stress protocol (Henze et al., 2017; Seddon et al., 2020). It consists of two tasks, a free speech and a mental arithmetic task, which are performed in front of a jury. Because our study required testing participants under pressure, we decided to employ the TSST to reliably induce stress, yet adjusted it to our setting. In particular, we used the original mental arithmetic task as the first task, followed by performing audition excerpts instead of the free speech in the original procedure.
