# Supplementary material for: Effects of tailored interventions for anxiety management in choking-susceptible performing artists: a mixed-methods collective case study
Source: Front Psychol. 2023 May 18;14:1164273. doi: 10.3389/fpsyg.2023.1164273 (PMC10232982; doi:10.3389/fpsyg.2023.1164273)
Supplement: Supplementary file 1 [file Table_1.DOCX]

# Supplementary Material

## Table S1. Overview of Participants’ Individual Audition Tasks.

| Pseudo-nym | Performance Major | Genre | Audition task | Duration (mm:ss) |
| --- | --- | --- | --- | --- |
| Anne | Dance | Contemporary | Choreography on *Data.Matrix* by Ryoji Ikeda | 03:30 |
| Bianca | Trumpet | Classical | Exposition of the first movement of the *Trumpet Concerto in E-flat major Hob. VIIe:1* by Joseph Haydn  Orchestra excerpt from the *Pulcinella Suite K. 043b* by Igor Stravinsky | 03:50 |
| Coco | Dance | Contemporary | Choreography on *Feel the Wind* by Johannes Beranek | 03:10 |
| Julia | Accordion | Classical | *Prelude and Fugue No. 9 in E major BWV 854* by Johann Sebastian Bach |  |
| Lucy | Acting | Stage Acting / Musical | Monologue from *Don Juan oder die Liebe zur Geometrie* by Max Frisch | 03:05 |
| Mia | Violin | Baroque | First structural part in major of the *Ciaconna* from the *Violin Partita No. 2 in D Minor BWV 1004* by Johann Sebastian Bach | 06:25 |
| Tom | Trumpet | Jazz | Improvisation on *Joyspring* by Clifford Brown | 03:40 |
| Vivi | Clarinet | Classical | Excerpts from the first and second movements of the *Clarinet Concerto in A major* K. 622 by Wolfgang Amadeus Mozart  Orchestra excerpt from the third movement of the *Symphony No. 9 in E-flat major op. 70* by Dmitri Shostakovich | 05:20 |
| Zoe | Voice | Jazz | Song *Nature Boy* by Nat King Cole | 03:30 |

*Note.* All musical excerpts were performed without accompaniment.
